# Circulating micrornas associated with glycemic impairment and progression in Asian Indians

**DOI:** 10.1186/s40364-015-0047-y

**Published:** 2015-10-13

**Authors:** Elena Flowers, Meghana Gadgil, Bradley E. Aouizerat, Alka M. Kanaya

**Affiliations:** Department of Physiological Nursing, School of Nursing, University of California, 2 Koret Way #N605L, CA 94143 San Francisco, USA; Division of General Internal Medicine, University of California, San Francisco, USA; Institute for Human Genetics, University of California, San Francisco, USA; Department of Epidemiology and Biostatistics, University of California, San Francisco, USA

**Keywords:** MicroRNA, Type 2 diabetes, Asian Indians, Glycemic impairment, Biomarkers

## Abstract

**Aims/hypothesis:**

Asian Indians have a high incidence of type 2 diabetes, but factors associated with glycemic progression in this population are not understood. MicroRNAs are emerging as important mediators of glucose homeostasis and have not been previously studied in Asian Indians. We examined microRNA (miR) expression associated with glycemic impairment and progression in Asian Indians from the San Francisco Bay Area. We studied 128 Asian Indians age 45–84 years without known cardiovascular disease and not taking diabetes medications. Oral glucose tolerance tests were performed at baseline and after 2.5 years. We quantified circulating miRs from plasma collected during the enrollment visit using a flow cytometry-based assay.

**Results:**

Glycemic impairment was present in 57 % (*n* = 73) at baseline. MiR-191 was positively associated with glycemic impairment (odds ratio (OR) 1.7 (95 % CI 1.2, 2.4), *p* < 0.01). The prevalence of glycemic progression after 2.5 years was 24 % (*n* = 23). Six miRs were negatively associated with glycemic progression: miR-122 (OR 0.5 (0.2, 0.8), *p* < 0.01), miR-15a (OR 0.6 (0.4, 0.9), *p* < 0.01), miR-197 (OR 0.6 (0.4, 0.9), *p* < 0.01), miR-320a (OR 0.6 (0.4, 0.9), *p* < 0.01), miR-423 (OR 0.6 (0.4, 0.9), *p* < 0.01), and miR-486 (OR 0.5 (0.3, 0.8), *p* < 0.01). Further multivariate adjustment did not attenuate these results.

**Conclusions/interpretation:**

This is the first study to investigate circulating miRs associated with glycemic status among this high-risk ethnic group. Individual miRs were significantly associated with both glycemic impairment and glycemic progression. Further studies are needed to determine whether miR (s) might be useful clinical biomarkers for incident T2D in the Asian Indian population.

**Electronic supplementary material:**

The online version of this article (doi:10.1186/s40364-015-0047-y) contains supplementary material, which is available to authorized users.

## Introduction

Asian Indians in the United States have a disproportionately higher prevalence (16–21 %) of type 2 diabetes mellitus (T2D) [1, 2] and associated metabolic conditions [3] compared to other racial groups. The exact biological mechanisms underlying risk in this population compared to other racial/ethnic groups are not well understood. Therefore approaches to risk reduction are limited and show suboptimal efficacy. Current risk screening approaches are limited to the detection of harmful metabolic conditions (i.e., impaired glucose tolerance, impaired fasting glucose). Identification of circulating biomarkers that provide insight about the underlying pathophysiology of T2D in Asian Indians, as well biomarkers associated with incident T2D prior to the onset of impaired glucose metabolism, may allow for earlier detection of the highest risk patients and improved treatments.

MicroRNAs (miRs) are components of an epigenetic mechanism regulating expression of messenger RNAs (mRNAs). Structurally, miRs are 18–24 nucleotide sequences that function by binding to a critical region on mRNA molecules in order to prevent translation into amino acids. More than 2,500 miRs have been identified in humans [4] and approximately 300 of these have been identified in the circulation from both cellular and non-cellular origins. Measurement of miRs captures combined genetic and environmental risk factors to provide a more comprehensive picture of the etiology of complex disease. Recent studies of primarily Caucasian non-Hispanic populations identified differential expression of circulating miRs in individuals with T2D compared to healthy controls [5–8]. MiR-126, which is active in endothelial cells, exhibits decreased expression in Caucasian individuals who develop T2D after 10 years compared to those who do not [8]. Cross-sectional studies of miR-126 identified lower expression in individuals with glycemic impairment or T2D. Other studies reported additional individual miRs (e.g., miR-140, miR-155 miR-221, miR-222, miR-320a, miR-375, miR-532) [9, 5, 10–12] that are differentially expressed in T2D and related conditions. These findings support the possibility that circulating miRs may provide novel insight about the biological mechanisms underlying T2D in Asian Indians and function as predictors of incident T2D. This study sought to (1) compare miR expression between Asian Indians with and without prevalent glycemic impairment; and (2) to compare miR expression between Asian Indians who had glycemic progression over 2.5 years to those who remained stable.

## Results

We studied 128 participants who were not taking diabetes medications at study enrollment. Of these, 57 % (*n* = 73) exhibited glycemic impairment (i.e., IGT, IFG, T2D). There are more males in this group (29 % vs. 18 %) and higher BMI (26.9 kg/m^2^ vs. 24.7 kg/m^2^) compared to individuals with normal glycemia (*p* < 0.01 for all, Table 1). The cardiovascular risk profile was worse in the impaired glycemia group with higher systolic (127 mmHg vs. 117 mmHg) and diastolic (75 mmHg vs. 69 mmHg) blood pressure, lower HDL-cholesterol (47 mg/dL vs. 53 mg/dL), and higher triglycerides (146 mg/dL vs. 115 mg/dL) (*p* < 0.05 for all, Table 1). There were no differences in total and LDL-cholesterol. Blood glucose (105 mg/dL vs. 87 mg/dL), insulin (15 μIU/mL vs. 10 μIU/ml), and HOMA-IR (4.1 μIU/mL*mmol/L vs. 2.2 μIU/mL*mmol/L) were all higher in the glycemic impairment group (*p* < 0.01 for all). Additional clinical characteristics were reported previously [13].

**Table 1:** Baseline demographic and clinical characteristics in individuals with and without glycemic impairment at baseline

Characteristics Mean ± SD, Median (IQR), or *n* (%)	Glycemic Impairment (*n* = 73)	Normal Glycemia (*n* = 55)	*p*-value
Sex (male)	29 (40)	18 (33)	0.002
Age (years)	56 ± 9	57 ± 7	0.897
Current Smokers	3 (4)	3 (5)	0.386
Physical Activity (MET-minutes/week)	1313 (585, 2940)	1365 (683, 2333)	0.942
Systolic Blood Pressure (mm Hg)	127 ± 15	117 ± 16	<0.001
Diastolic Blood Pressure (mm Hg)	75 ± 12	69 ± 9	0.002
Total Cholesterol (mg/dL)	191 ± 33	194 ± 31	0.683
LDL-c (mg/dL)	116 ± 31	117 ± 27	0.731
HDL-c (md/dL)	47 ± 13	53 ± 15	0.016
Triglycerides (mg/dL)	146 ± 72	115 ± 39	0.006
Fasting Blood Glucose (mg/dL)	105 ± 21	87 ± 6	<0.001
Insulin (μIU/mL)	15 ± 11	10 ± 5	0.001
HOMA-IR (μIU/mL*mmol/L)	4.1 ± 3.4	2.2 ± 1.1	<0.001
Body Mass Index (kg/m^2^)	26.9 ± 5.1	24.7 ± 3.6	0.006
Waist Circumference (cm)	98 ± 13	91 ± 11	0.003

*cm* centimeters, *HDL-c* high density lipoprotein cholesterol, *HOMA-IR* homeostasis model assessment-insulin resistance, *kg/m* ^*2*^ kg per meter^2^, *LDL-c* low density lipoprotein cholesterol, *mcU/mL* microUnit per milliliter, *mg/dL* milligrams per deciliter, *mm Hg* millimeters of mercury, *SD* standard deviation

Of the 94 participants evaluated after 2.5 years, 24 % (*n* = 23) exhibited glycemic progression. There were no statistically significant differences in baseline clinical characteristics between those who did and did not have incident glycemic impairment (Table 2). In paired analyses, we observed that blood glucose (91 mg/dL vs. 101 mg/dL), insulin (11.5 μIU/mL vs. 17.8 μIU/ml), and HOMA-IR (2.6 μIU/mL*mmol/L vs. 4.5 μIU/mL*mmol/L) increased after 2.5 years in the subset of participants (*n* = 23) whose glycemic impairment worsened (Table 3). However there were no significant changes in cardiovascular risk factors, waist circumference, or BMI.

**Table 2:** Baseline demographic and clinical characteristics in individuals with progressive glycemic impairment compared to no change at 2.5 years

Characteristics Mean ± SD, Median (IQR), or *n* (%)	Progressive Glycemic Impairment (*n* = 23)	No Change (*n* = 71)	*p*-value
Sex (male)	14 (61)	41 (58)	0.070
Age (years)	55 ± 9	57 ± 9	0.310
Current Smoking	8 (9)	3 (4)	0.705
Physical Activity (MET-minutes/week)	1260 (473, 2100)	1470 (613, 2940)	0.293
Systolic Blood Pressure (mm Hg)	119 ± 20	122 ± 17	0.420
Diastolic Blood Pressure (mm Hg)	71 ± 13	71 ± 10	0.942
Total Cholesterol (mg/dL)	188 ± 39	188 ± 29	0.955
LDL-c (mg/dL)	113 ± 33	112 ± 27	0.872
HDL-c (md/dL)	51 ± 16	50 ± 14	0.780
Triglycerides (mg/dL)	120 ± 65	126 ± 56	0.676
Fasting Blood Glucose (mg/dL)	91 ± 8	91 ± 11	0.813
Insulin (μIU/mL)	11.5 ± 6.2	11.5 ± 6.2	0.807
HOMA-IR (μIU/mL*mmol/L)	2.6 ± 1.5	2.8 ± 2.1	0.715
Body Mass Index (kg/m^2^)	25.7 ± 4.7	25.9 ± 4.5	0.878
Waist Circumference (cm)	93 ± 12	94 ± 12	0.856

*cm* centimeters, *HDL-c* high density lipoprotein cholesterol, *HOMA-IR* homeostasis model assessment-insulin resistance, *kg/m* ^*2*^ kg per meter^2^, *LDL-c* low density lipoprotein cholesterol, *mcU/mL* microUnit per milliliter, *mg/dL* milligrams per deciliter, *mm Hg* millimeters of mercury, *SD* standard deviation

**Table 3:** Change in clinical characteristics in individuals with progressive glycemic impairment after 2.5 years (*n* = 23)

Characteristics Mean ± SD or *n* (%)	Baseline	Follow up (2.5 Years)	*p*-value
Systolic Blood Pressure (mm Hg)	119 ± 20	123 ± 13	0.235
Diastolic Blood Pressure (mm Hg)	71 ± 13	76 ± 13	0.081
Total Cholesterol (mg/dL)	188 ± 39	184 ± 30	0.364
LDL-c (mg/dL)	113 ± 33	106 ± 26	0.091
HDL-c (md/dL)	51 ± 16	51 ± 20	0.895
Triglycerides (mg/dL)	120 ± 65	137 ± 69	0.211
Fasting Blood Glucose (mg/dL)	91 ± 8	101 ± 18	0.008
Insulin (μIU/mL)	11.5 ± 6.2	17.8 ± 6.6	<0.001
HOMA-IR (μIU/mL*mmol/L)	2.6 ± 1.5	4.5 ± 2.1	<0.001
Body Mass Index (kg/m^2^)	25.7 ± 4.7	26.3 ± 4.9	0.065
Waist Circumference (cm)	93 ± 12	94 ± 13	0.796

*cm* centimeters, *HDL-c* high density lipoprotein cholesterol, *HOMA-IR* homeostasis model assessment-insulin resistance, *kg/m* ^*2*^ kg per meter^2^, *LDL-c* low density lipoprotein cholesterol, *mcU/mL* microUnit per milliliter, *mg/dL* milligrams per deciliter, *mm Hg* millimeters of mercury, *SD* standard deviation

Of the thirty miRs measured, 6 (i.e., miR-138, miR-192, miR-193b, miR-214, miR-370, miR-375) were below the limit of detection (i.e., 10 AU) and failed to retain a minimum of at least of two of three technical replicates for every participant and were excluded from the analysis. One of the remaining 24 miRs was significantly associated with glycemic impairment at baseline. In unadjusted logistic regression models, miR-191 (odds ratio (OR) 1.7 (1.2, 2.4)) was positively associated with glycemic impairment (*p* < 0.05) (Table 4). In a multivariate adjusted model including age, sex, BMI, and waist circumference, the odds ratio remained statistically significant. Further adjustment for HDL-cholesterol, triglycerides, and blood pressure did not attenuate these effects.

**Table 4:** Odds ratios for microRNA expression associated with glycemic impairment

	Unadjusted			Model 1*			Model 2**		
	OR^€^	95 % CI	*p*-value	OR^€^	95 % CI	*p*-value	OR^€^	95 % CI	*p*-value
Prevalent Glycemic Impairment									
hsa-miR-191-5p	1.7	1.2, 2.4	0.002	1.6	1.1, 2.3	0.014	1.5	1.1, 2.2	0.026
Progressive Glycemic Impairment After 2.5 Years									
hsa-miR-122-5p	0.5	0.2, 0.8	0.003	0.4	0.3, 0.8	0.003	0.5	0.3, 0.8	0.003
hsa-miR-15a-5p	0.6	0.4, 0.9	0.040	0.6	0.4, 0.9	0.031	0.6	0.4, 0.9	0.034
hsa-miR-197-3p	0.6	0.4, 0.9	0.020	0.6	0.4, 0.9	0.016	0.6	0.4, 0.9	0.018
hsa-miR-320a	0.6	0.4, 0.9	0.038	0.6	0.4, 0.9	0.045	0.6	0.4, 0.9	0.032
hsa-miR-423-5p	0.6	0.4, 0.9	0.033	0.6	0.4, 0.9	0.039	0.6	0.4, 0.9	0.030
hsa-miR-486-5p	0.5	0.3, 0.8	0.004	0.4	0.3, 0.8	0.003	0.4	0.3, 0.8	0.003

*CI* confidence interval, *MET* metabolic equivalent

^€^microRNAs are scaled by quartile

*Model 1 adjusted for age, sex, and waist circumference

**Model 2 adjusted for variables in Model 1 and HDL-cholesterol and triglycerides

Six miRs were significantly associated with glycemic progression after 2.5 years. These included miR-122 (odds ratio (OR) 0.5 (95 % CI 0.2, 0.8)), miR-15a (OR 0.6 (0.4, 0.9)), miR-197 (OR 0.6 (0.4, 0.9)), miR-320a (OR 0.6 (0.4, 0.9)), miR-423 (OR 0.6 (0.4, 0.9)), and miR-486 (OR 0.5 (0.3, 0.8)), which were all inversely associated with glycemic progression (*p* < 0.01 for all) (Table 4). We adjusted for age, sex, and waist circumference and observed the same significant association with glycemic progression. We then added HDL-cholesterol, triglycerides, and blood pressure to the model and observed the same significant associations. Fold changes are presented in Additional file 1: Table S1.

## Discussion

To our knowledge, this is the first study to investigate circulating miRs associated with glycemic impairment in the high risk Asian Indian ethnic group. We evaluated the association between circulating miRs and both prevalent glycemic impairment and glycemic progression after 2.5 years. We found a positive relationship between miR-191 and the presence of glycemic impairment. Six miRs (i.e., miR-122, miR-15a, miR-197, miR-320a, miR-423, miR-486) were differentially expressed between the subset of individuals who had incident glycemic impairment after 2.5 years compared to those who remained stable. All six miRs exhibited an inverse relationship with odds for glycemic progression. The addition of clinical covariates (i.e., age, sex, waist circumference, HDL-cholesterol, triglycerides) to logistic regression models did not meaningfully attenuate the association for either glycemic impairment or glycemic progression.

Asian Indians living in the United States have a high prevalence of T2D and related risk factors [3, 14, 1] and the prevalence is increasing drastically in India and other parts of South Asia [15, 16]. While there are some known common risk factors for T2D in this population (i.e., diet, sedentary lifestyle, genetic predisposition), the full etiology is not well understood and therefore approaches to risk reduction have been limited. Identification of circulating miRs associated with glycemic impairment has the potential to provide novel information about the mechanisms underlying T2D. Further, there is the possibility for detection of homogeneous subgroups of individuals at risk based on commonalities in miR expression. In addition, miRs may be useful biomarkers to identify individuals at highest risk for developing T2D who might benefit from aggressive prevention measures.

MiR-486 is inversely associated with risk for glycemic progression. We previously identified that this miR is positively associated with insulin resistance and response to thiazolidenidones, which improve insulin sensitivity, in a multiracial sample [17]. MiR-486 is enriched in muscle tissue where it targets phosphatase and tensin homolog (PTEN) and forkhead box O1a (FOXO1A) and regulates muscle growth and atrophy in mice [18, 19]. In other tissues, PTEN is implicated in insulin signaling and cellular glucose uptake. Inhibition of PTEN decreases blood glucose in rats [20] and mutations in PTEN in humans are associated with insulin sensitivity and glucose tolerance [21]. Peroxisome proliferator-activated receptor γ (PPAR- γ), the primary target of thiazolidenidones, upregulates PTEN, which may lead to changes in insulin sensitivity and blood glucose [22].

MiR-486 has also been implicated in other diseases. Findings from these studies implicate potential mRNAs and biologic pathways targeted by miR-486 that are relevant to a regulatory role in development of risk for T2D. In lung cancer, miR-486 regulates the insulin growth factor signaling pathway by targeting insulin-like growth factor 1 (IGF1), IGF1 receptor (IGF1R), and phosphoinositide-3-kinase, regulatory subunit 1 (alpha) (PIK3R1) [23]. Two prior studies identified low levels of miR-486 in hepatocellular carcinoma cells (HCC) from human biopsy samples and HCC lines *in vitro*. Functional targets of miR-486 in HCC include PIK3R1 [24] and rho-interacting serine/threonine kinase (CITRON) and claudin 10 (CLDN10) [25]. MiR-122, which was negatively associated with glycemic progression in our study and appears to be a target of PPAR- γ [26], is the primary hepatic miR and regulates progression of HCC [27]. Whether miR-486 and miR-122 are co-expressed in hepatocytes and may have related functions in insulin and glucose metabolism in liver or other tissues is not known. Further functional studies are needed to determine if the pathways regulated by miR-486 in other diseases might also be relevant to the development of T2D.

In addition to HCC, associations between miR-122 and other hepatic diseases, including hepatitis, have been well described [28, 29]. Data from *in vitro* studies of hepatic cell lines provide evidence for regulation of hepatic lipid metabolism by miR-122 [30], however our prior study [31] and others [11] found a low level of this miR in individuals free from serious hepatic disease using quantitative polymerase chain reaction-based quantitation.

We found that miR-15 was decreased in individuals who had glycemic progression after 2.5 years. MiR-15a was also decreased in the study of prevalent T2D after 10 years in a Caucasian sample [8]. One *in vitro* study found that miR-15 is increased after 1-h of exposure to glucose but decreased with long-term exposure and may mediate B-cell function and insulin biosynthesis by targeting uncoupling protein-2 (UCP-2) [32]. Several additional studies support a critical role of miR-15a in endothelial cell function and angiogenesis in peripheral vascular, myocardial, and cerebrovascular tissue [33–36]. MiR-15 is increased in the serum of diabetic patients with critical limb ischemia and decreases angiogenesis in pro-angiogenic cells *in vitro* by targeting vascular endothelial growth factor A (VEGFA) [34]. Increased levels of miR-15 are associated with myocardial ischemia/reperfusion injury in mice [33] and suppression of miR-15 by PPAR-γ in cerebral vascular endothelial cells increases pro-angiogenic activity in animal models and *in vitro* studies [35]. We found decreased expression of miR-15 prior to the onset of T2D, which corresponds with pro-angiogenic activity according to data from functional studies. Further research is needed to determine whether there are mechanisms to protect from the long-term vascular consequences of T2D early in the course of the disease.

Consistent with our findings, one prior study identified decreased miR-423 in individuals with T2D and or obesity compared to normal weight non-diabetics [5]. In the same study, neither insulin or treatment with metformin and associated weight loss were associated with any changes in miR-423, though this miR is highly correlated with measures of insulin sensitivity [5]. Only one other study reporting significant findings for miR-423 was identified. In an *in vitro* model of HCC, miR-423 is up-regulated and targets the tumor suppressor gene p21Cip1/Waf1 facilitating oncogenesis [37].

MiR-320, which was decreased in our study, appears to also be enriched in endothelial cells and respond to oxidative stress to regulate cell migration and proliferation [38–41]. Predicted targets of miR-320a from functional studies include IGF1R, polycomb ring finger oncogene (BMI-1), heme-oxygenase 1 (HO-1), glutathione cysteine ligase modifying subunit (GCLM) and oxidative stress induced growth inhibitor 1 (OKL38) and VEGF. Up-regulation of miR-320a results in decreased negative responses to oxidative stress [40, 41] and decreased cellular migration and tumor invasion [38, 39].

Little is known about the function of miR-197. This miR is computationally predicted to target several hundred possible mRNAs, including targets associated with type 2 diabetes (e.g., glucagon (GCG), deiodinase, iodothyronine, type II (DIO2), leptin (LEP), NK2 homeobox 2 (NKX2-2), solute carrier family 30, member 8 (SLC30A8), synapsin I (SYN1), nuclear respiratory factor 1 (NRF1), SHC transforming protein 1 (SHC1), Kruppel-like factor 12 (KLF12), protein kinase C, beta (PRKCB) [42, 43]. Further studies are needed to refine the list of possible mRNA targets and determine the biologic pathways regulated by this miR.

MiR-320a and miR-486 were decreased in individuals who exhibited glycemic progression after 2.5 years. By contrast, our prior study showed a direct relationship with prevalent insulin resistance [17]. The apparent discrepancy in these findings may be the result of the time point at which miRs were measured relative to the progression of disease. In the prior study, we included individuals with confirmed insulin resistance but no diabetes, whereas in this study, we compared individuals who were further along the glycemic spectrum than having isolated insulin resistance without any glycemic impairment. A recent meta-analysis of miRs associated with type 2 diabetes identified similar inconsistencies between studies [11]. Future longitudinal studies relating changes in miRs and progression to T2D are needed in order to understand whether individual miRs are the cause or the consequence of glycemic impairment. A second possible explanation is that our prior study included a multi-racial sample, whereas this study included only Asian Indians.

MiR-126 was associated with risk for type 2 diabetes after 10-years in a primarily Caucasian sample [8], but in our study this miR was one of the least variable and was selected as a normalizer. Similarly to above, possible explanations include the time course of disease compared to when the microRNAs were measured and differences in disease mechanisms between Asian Indians and other racial groups.

The specific biologic mechanisms underlying glycemic impairment and T2D vary between groups and individuals. Identification of circulating miR biomarkers has the possibility to provide information about the specific causal pathways for an individual or group of individuals. Functional studies are needed to identify and validate the exact mRNA targets of the miRs that are differentially expressed in Asian Indians with prevalent or incident glycemic impairment compared to healthy individuals. Future clinical implications include optimization of treatments to target relevant biologic pathways and identification of new pharmacologic targets. Further studies with larger sample sizes and longer follow up are needed to validate the findings for miRs reported here and determine whether there is a set of miRs that significantly improve prodromal risk prediction. Finally, comparisons of circulating miR expression between Asian Indians and other racial groups will provide insight into similarities and differences in the etiology of T2D with future implications for treatments.

There are limitations to this study worthy of consideration. Standardized approaches to normalization of miR expression data from plasma and serum samples are not established. However, consensus is building around normalization approaches that average signals from multiple miRs [44–47]. We used the geNorm algorithm to select the set of three miRs that showed low variability in our sample. These included miR-126, miR-21, and miR-24. Interestingly, miR-126, which showed very little variability in our sample, was previously associated with risk for T2D after 10 years in a primarily Caucasian sample [8]. In a prior study, we showed that MiR-21 is associated with atherogenic dyslipidemia in South Asian men [31]. This miR is also well established in inflammatory pathways [48]. The incongruity of our findings compared with previous studies supports the need to establish standards for data normalization and other methodologic considerations in order to compare findings between studies. Another limitation of this study is the selection of the subset of miRs for which there was some *a priori* evidence to suggest a functional role in glucose metabolism. Future studies should incorporate an agnostic approach to miR detection in order to identify novel targets related to risk for T2D.

We identified individual circulating miRs associated with prevalent glycemic impairment and glycemic progression in the high risk Asian Indian population. Among these are several miRs that have previously been associated with T2D and related conditions in other studies, as well as miRs that are predicted to be implicated in biologically relevant pathways. This is promising evidence to suggest that circulating miRs may be useful for identification of the biologic mechanisms underlying T2D in Asian Indians. Implications include optimization of treatment approaches and new treatment targets. Additional potential clinical applications include improved detection of glycemic impairment and identification of individuals at high risk for progression to T2D.

## Methods

### Study design, sample, and clinical data collection

We studied participants in the Metabolic syndrome in Asian Indians Living in America (MASALA) Study [13]. Briefly, this is a sample of community-dwelling adults who self-identify as Asian Indian and were free from known cardiovascular disease at baseline. Of 150 participants in the MASALA study, 149 provided complete baseline data. We excluded 21 participants taking diabetes medications at baseline for a sample size for evaluation of prevalent glycemic impairment of 128. After 2.5 years of follow-up, 112 participants completed a second clinical examination. Of these, we excluded 18 participants who had T2D at baseline for a sample size of 94 in the follow-up visit. Participants were sampled by surname from all counties in the San Francisco Bay Area as described previously [13]. Demographic information was collected by self-report. Height, weight, waist circumference, and blood pressure were collected by trained study personnel [13]. Physical activity was assessed using the Typical Week’s Physical Activity Questionnaire [49]. Participants were administered 75 g oral glucose and blood glucose measurements were obtained after 120 min. Impaired glucose tolerance (IGT) was defined as 2-h glucose 140–199 mg/dl, impaired fasting glucose (IFG) was defined as fasting glucose 100–125 mg/dl, and T2D was defined as 2-h glucose ≥200 mg/dl, or fasting glucose ≥126 mg/dl. Glycemic impairment was defined as the presence of IGT, IFG, or T2D at baseline. Glycemic progression was defined as (1) progression from normal glycemia to IGT, IFT, or T2D; (2) from IGT to IFG or T2D; or (3) from IFG to T2D after 2.5 years of follow-up. Body mass index (BMI) was calculated using the formula kilograms/meters^2^. To estimate insulin sensitivity we calculated homeostasis –model assessment of insulin resistance (HOMA-IR) using the formula I_0_ (μIU/mL) * G_0_ (mmol/L)/22.5 [50]. Blood used for banking of plasma was collected at baseline into vacutainers containing the preservative EDTA, centrifuged at 4 °C to separate plasma from cellular blood components, and stored at −80 °C. The study was approved by the University of California, San Francisco Institutional Review Board.

### Molecular data collection

We selected a panel of 28 miRs (i.e., miR-122-5p, miR-126-3p, miR-138-5p, miR-140-5p, miR-146a-5p, miR-146b-5p, miR-15a-5p, miR-150-5p, miR-191-5p, miR-192-5p, miR-193b-3p, miR-195-5p, miR-197-3p, miR-20b-5p, miR-21-59, miR-222-3p, miR-223-3p, miR-24-3p, miR-27a-3p, miR-29b-3p, miR-320a, miR-33a-3p, miR-33b-5p, miR-370-3p, miR-375, miR-423-5p, miR-486-5p, miR-503-5p) to measure based on previous data from human studies of blood-based expression of miRs in T2D and related conditions. Two miRs (i.e., miR-214-3p, miR-22-3p) were selected as potential normalizers. MicroRNAs were quantified using the Firefly Circulating miRNA Assay (Firefly BioWorks, Cambridge, MA) providing direct detection of microRNAs from plasma without isolation [51]. MiRs from plasma were hybridized to complementary oligonucleotides covalently attached to encoded hydrogel microparticles. The bound target was ligated to oligonucleotide adapter sequences that serve as universal PCR priming sites. The miR-adapter hybrid molecules were then denatured from the particles at 95 °C and reverse transcription polymerase chain reaction (RT-PCR) was performed using a fluorescent forward primer. Once amplified, the fluorescent target was rehybridized to the original capture particles and scanned on a EMD Millipore Guava 8HT Flow Cytometer (Merck KGaA Darmstadt, Germany). This assay exhibits 90–92 % concordance with other quantitation assays [52] and has been used in previous studies published in peer-reviewed journals [53–55, 17]. Three technical replicate measurements were obtained for each sample and miR target. MiRs that failed to retain at least two of three technical replicates with a robust enough signal for analysis (i.e., 10 AU) for each sample were excluded from analysis.

### Statistical analysis

Student’s *t*-tests, Pearson’s chi-squared tests, and Wilcoxon rank sum tests were used to compare demographic characteristics between groups and paired samples *t*-test were used to compare the sample over time. Normalized expression of each sample for each miR target was calculated using the geometric mean expression of three miRs (i.e., miR-126-3p, miR-21-5p, miR-24-3p) selected using the geNorm algorithm [45]. Logistic regression models were used to determine odds ratios with sequential adjustment for covariates. MiRs were log transformed for logistic regression models. Fold change differential expression of miRs between groups was calculated as the ratio of normalized expression in the group with prevalent glycemic impairment or glycemic progression compared to those who were normoglycemic or stable, respectively. Wilcoxon rank sum tests were used to compare normalized expression of miRs between groups (α = 0.05). We used a permutation test to control for the family-wise error rate (FWER) across all miRs.

## Additional file

Additional file 1: Table S1. Fold Change MicroRNA Expression. (DOCX 26 kb)

## Abbreviations

miR: MicroRNA
T2D: Type 2 diabetes
