# Supplementary material for: Circulating micrornas associated with glycemic impairment and progression in Asian Indians
Source: Biomark Res. 2015 Oct 13;3:22. doi: 10.1186/s40364-015-0047-y (PMC4785747; doi:10.1186/s40364-015-0047-y)
Supplement: Additional file 1: Table S1. — Fold Change MicroRNA Expression. (DOCX 26 kb) [file 40364_2015_47_MOESM1_ESM.docx]

**Table S1.** Fold Change MicroRNA Expression

|  | **Glycemic Impairment (n=73) vs.**  **Normal Glycemia (n=55)**  **at Baseline** | | | **Progressive Glycemic Impairment (n=23) vs. No Change (n=71)  After 2.5 Years** | | |
| --- | --- | --- | --- | --- | --- | --- |
|  | **Fold Change** | **p-value*** | **p-value**** | **Fold Change** | **p-value*** | **p-value**** |
| miR-122-5p | 1.12 | 0.43 | 0.43 | 0.51 | 0.16 | 0.17 |
| miR-126-3p | 1.01 | 0.52 | 0.52 | 1.01 | 0.14 | 0.14 |
| miR-140-5p | 0.89 | 0.88 | 0.87 | 1.06 | 0.62 | 0.62 |
| miR-146a-5p | 0.96 | 0.35 | 0.35 | 1.10 | 0.39 | 0.39 |
| miR-146b-5p | 0.97 | 0.49 | 0.49 | 1.13 | 0.79 | 0.80 |
| miR-15a-5p | 0.98 | 0.65 | 0.65 | 0.80 | 0.45 | 0.45 |
| miR-150-5p | 1.03 | 0.78 | 0.78 | 0.70 | 0.07 | 0.07 |
| miR-191-5p | 1.06 | 0.85 | 0.85 | 0.97 | 0.43 | 0.43 |
| miR-195-5p | 0.97 | 0.84 | 0.84 | 0.88 | 0.81 | 0.81 |
| miR-197-3p | 1.09 | 0.95 | 0.95 | 0.81 | 0.84 | 0.85 |
| miR-20b-5p | 0.92 | 0.19 | 0.19 | 0.85 | 0.64 | 0.64 |
| miR-21-5p | 0.96 | **0.02** | **0.02** | 1.01 | 0.81 | 0.82 |
| miR-22-3p | 1.01 | 0.37 | 0.37 | 0.86 | 0.86 | 0.98 |
| miR-222-3p | 1.01 | 0.89 | 0.90 | 1.05 | 0.72 | 0.72 |
| miR-223-3p | 1.02 | 0.39 | 0.39 | 0.94 | 0.33 | 0.32 |
| miR-24-3p | 1.01 | 0.26 | 0.26 | 1.00 | 0.12 | 0.12 |
| miR-27a-3p | 0.98 | 0.09 | 0.10 | 1.02 | 0.56 | 0.56 |
| miR-29b-3p | 0.98 | 0.16 | 0.16 | 0.87 | 0.32 | 0.32 |
| miR-320a | 1.01 | 0.46 | 0.46 | 0.89 | 0.88 | 0.88 |
| miR-33a-5p | 0.98 | 0.26 | 0.27 | 1.09 | 0.33 | 0.33 |
| miR-33b-5p | 0.89 | 0.47 | 0.47 | 1.36 | 0.95 | 0.95 |
| miR-423-5p | 1.01 | 0.56 | 0.56 | 0.81 | 0.79 | 0.79 |
| miR-486-5p | 1.03 | 0.14 | 0.14 | 0.57 | 0.92 | 0.92 |
| miR-503-5p | 0.96 | 0.57 | 0.56 | 1.04 | 0.37 | 0.37 |

*Wilcoxon rank sum test

**adjusted p-value using permutation tests

Family-wise error rate Permutation corrected p-value is 0.27 for prevalent glycemic impairment and 0.73 for glycemic progression.
